# Does Optimization of Industrial Structure Improve Green Efficiency of Industrial Land Use in China?

**DOI:** 10.3390/ijerph19159177

**Published:** 2022-07-27

**Authors:** Bingqing Li, Zhanqi Wang, Feng Xu

**Affiliations:** School of Public Administration, China University of Geosciences, Wuhan 430074, China; libingqing@cug.edu.cn (B.L.); whcugxf@cug.edu.cn (F.X.)

**Keywords:** industrial land use, green efficiency, industrial structure, spatial effects, spatial Durbin model, China

## Abstract

Improving the green efficiency of industrial land use (GEILU) is essential to promoting low-pollution and highly efficient development, and industrial structure is a key factor in this dynamic. This paper aims to reveal how the optimization of industrial structure (OIS) affects GEILU in China. First, an analytical framework was proposed to understand the effect mechanisms from the perspective of rationalization, upgrading, and ecologization of industrial structure. Second, the panel data of 31 provincial units collected from 2006 to 2020 were taken as cases for empirical study. The super-SBM model was adopted to measure GEILU, and some variables were used to evaluate OIS. Finally, the spatial effects of OIS on GEILU were analyzed based on the spatial Durbin model. The results show that the GEILU presented a wave change and kept increasing after 2016. From a global perspective, the rationalization of industrial structure helped improve GEILU; however, the upgrading and ecologization of industrial structure generated inhibiting effects. When integrating the three perspectives, optimization of industrial structure was considered to have negative effects on GEILU. The negative effects stemmed from an inefficient expansion of industrial land and pollution from heavy chemical industries. From a phased perspective, in the early period of this study, the outdated technology in traditional industries brought about the negative effects; however, with high-knowledge and high-tech industries forming a market scale, optimization of industrial structure gradually became conducive to the improvement of GEILU. This study suggests that eliminating the market segmentation between provinces and accelerating the development of high-knowledge and high-tech industries can help promote low-pollution and highly efficient industrial land use in China.

## 1. Introduction

China has become the largest manufacturing country and formed the most complete industrial system in the world [1]. Continuous supply of large-scale industrial land is one of important driving forces in this process [2,3]. From 2006 to 2019, urban industrial land area in China increased from 6.9 × 10^5^ ha to 1.1 × 10^6^ ha, while the total industrial output grew from 1.0 × 10^4^ billion to 3.2 × 10^4^ billion. However, low-density industrial land development and high industrial-pollution emissions have seriously threatened sustainable development and sparked widespread concern across the nation [4]. In comparison, the proportion of industrial land to built-up land area in most developed countries is about half of that in China, but the utilization efficiency of industrial land is much higher than that of China [1]. Industrial enterprises are still an important source of environmental pollution [5]. Within this context, the Chinese government prioritized the low-pollution and highly efficient utilization of land resources [6,7]. The report delivered at the 19th National Congress of the Communist Party of China emphasized the strategy of high-quality development; highlighted an adherence to conservation, protection, and natural recovery efforts; and stressed the importance of creating a production mode that conserves resources and protects the environment.

**Figure 1:**
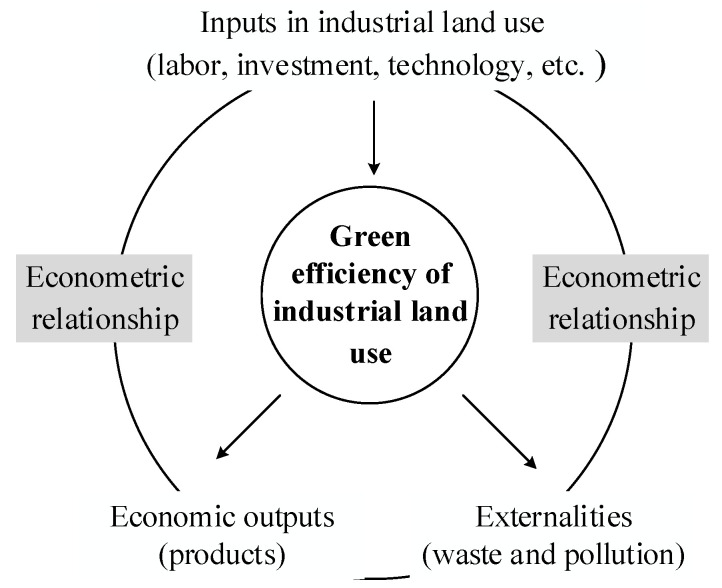
Formation of the green efficiency of industrial land use.

Under the requirements of sustainable development, green efficiency has become an important index to industrial land management, especially in developing countries such as China [8,9,10,11,12,13]. Although China is a geographically large country, per capita land area is far below the world average. After a rapid urban land expansion, the supply of newly added industrial land in cities is tightening in recent years [14]. For ensuring economic development, many policies have been introduced by the government to improve industrial land-use efficiency, such as promoting inefficient land redevelopment, stipulating the standard of industrial land-use intensity, etc. [15,16]. At first, industrial land-use efficiency emphasizes economic utilization of land resources and refers to the output level of industrial land under a certain input, where the output usually is the value of products generated by enterprises using the land, and the input includes factors of production such as labor, investment, and technology [17]. Later, it is realized that industrial production generates expected output for economy but at the same time produces waste and pollution, that is, negative externalities [18]. In view of this, scholars gradually pay attention to the green efficiency of industrial land use (GEILU) [19]. GEILU can be defined as a synthetic level of economic output and eco-environmental impacts obtained through certain inputs on industrial land (Figure 1). It has the advantage of explaining both economical utilization of land resources and the disturbance to eco-environment [20]. GEILU is achieved by gaining the most goods and services in ways that reduce ecological disturbance and land consumption. It is often measured by analyzing the econometric relationship between land input factors, industrial output values, and some negative externalities of environmental pollution, such as harmful gas emissions, solid waste emissions, and soot emissions. The index system method, some extended models based on data envelopment analysis, and stochastic frontier analysis are used to quantify this econometric relationship [21,22,23].

Industrial structure refers to the composition of industrial categories in regional industrial development and is usually evaluated by integrating the distribution of production factors and proportion of output value among industrial categories [24]. It is a key factor in the process of changing GEILU via affecting economical utilization of industrial land and the eco-environmental disturbance [25,26,27,28]. First, industrial enterprises differ with respect to their returns on land investment. For example, in China, high-tech and high-economic-input industries are believed to exhibit higher intensity of land use and higher return on investment. Second, industrial enterprises also differ with respect to their production of pollutants. For instance, chemical industries and mining industries have greater ecological risks. Under the strategy of high-quality development, the Chinese government believes that the optimization of industrial structure (OIS) is extremely important and views it as one of the main measures to promote low-pollution and highly efficient utilization of resources. It is expected that GEILU will be improved with OIS.

For the above issue, some scholars have studied the relationship between OIS and land use. Caldarelli and Gilio (2018) analyzed the expansion of sugarcane industry and found that it impacted land-use patterns and employment trends in several of Brazil’s regions [29]. Yang et al. (2019) explored the relationship between land-use structure and industrial structure [30]. Liu et al. (2021) discovered a complex non-linearity and spatial relationship between OIS and urban land-use efficiency [24]. Zhang et al. (2019) proved that the specialization, diversification, and upgrading of industrial structure increased the proportion of high-knowledge and high-tech industries within the structure and produced external effects on land use, thus improving highly efficient urban land use [31]. Existing studies have established a connection between industrial structure and land use based on the industrial development policies of various countries. Chinese scholars pay great attention to urban land-use efficiency with the development of high-tech industries and transformation and upgrading of industrial structure. However, there is still a lack of systematic theoretical analysis on the effect mechanisms of OIS on GEILU. Moreover, they often integrate all types of land in urban area as the research object while exploring the relationship between urban land-use efficiency and industrial structure rather than separating industrial land from urban land and analyzing the relationship between industrial land-use efficiency and the corresponding industrial structure. Therefore, there is a lack of special empirical research on the effects of OIS on GEILU. What are the effects of OIS on GEILU? How does these effects occur? If there is a positive effect, it is an expected result conducive to the high-quality development. If there is a negative effect, it indicates that the industrial land use or industrial structure needs to be further optimized.

For these reasons, this paper focuses on industrial land use while aiming to reveal how OIS affects GEILU in China. The panel data of 31 provincial units collected from 2006 to 2020 were taken as cases for empirical study. The super-SBM model was adopted to measure GEILU, and some variables were used to evaluate OIS. Finally, the spatial effects of OIS on GEILU were analyzed based on the spatial Durbin model. Ultimately, this study deepens the understanding of China’s GEILU and provides a reference for improving GEILU. This study contributes to the existing literature in several ways. First, it proposes an analytical framework to understand the effect mechanisms of OIS on GEILU, which stresses the need to take into account rationalization, upgrading, and ecologization of industrial structure. This analysis framework can provide a theoretical basis for subsequent empirical studies. Second, this study strongly indicates the presence of spatial effects of OIS on GEILU in China. Finally, we prove that there were obviously threshold effects, which shows the direction of effects were related to the stage of industrial development. That is, with the optimization of industrial structure from heavy-chemical industry to high-tech industry, the effects gradually shifted from negative into positive.

## 2. Analytical Framework of the Effect Mechanisms

**Figure 2:**
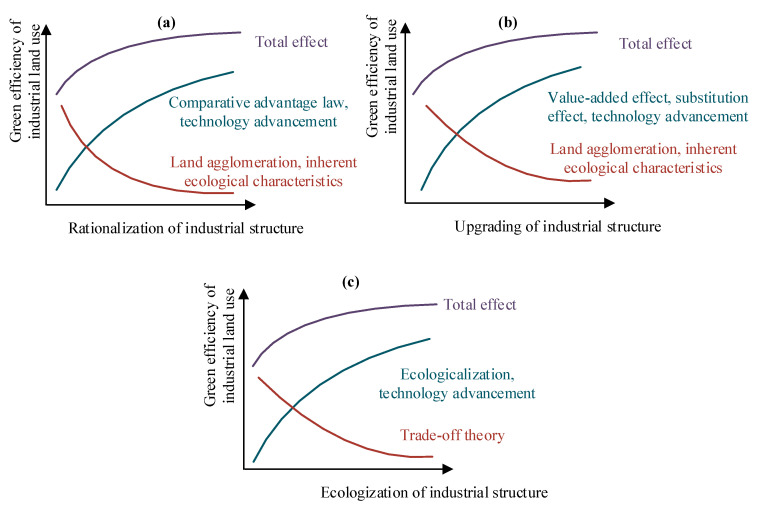
The effect mechanisms of industrial structure optimization on green efficiency of industrial land use. (**a**–**c**) show the effect mechanisms of RIS, UIS, and EIS on GEILU, respectively.

In reference to the theory of industrial structure, there are three common variables for explaining the OIS: (1) rationalization of industrial structure (RIS), (2) upgrading of industrial structure (UIS), and (3) ecologization of industrial structure (EIS) [23,32,33]. RIS refers to the coordinated development of regional industries through the rational allocation of production factors in various industrial categories. UIS is a process that involves developing industries with high factor productivity and eliminating industries with low factor productivity. EIS is a process that involves developing industries with low consumption, low pollution, and low emissions. This section theoretically analyzes how OIS changes GEILU using the three progressive levels of rationalization, upgrading, and ecologization (Figure 2). Figure 2a–c shows the effect mechanisms of RIS, UIS, and EIS on GEILU, respectively.

### 2.1. Rationalization of Industrial Structure

RIS has both positive and negative effects on GEILU. The positive effects are generated through comparative advantage law and technology advancement [32]. In terms of comparative advantage law, RIS can fully tap into the potential productivity of land by improving the enterprises that use the land. Under the guidance of market mechanism, industrial land with extensive use and low value-added ratios will cease production and then be leased to other enterprises that suit local science and technology levels, consumption demands, population characteristics, and resource conditions [31,33,34]. As a result, enterprises gather in land circles that are suitable for productivity, which can inspire the comparative advantages of land use and increase the output of industrial land. In terms of technology advancement, the economy development drives the innovation of governance concept and introduction of advanced technology. Enterprises will seek more profits through the reuse of industrial waste and reducing pollutant production. In this process, disturbance to the eco-environment will decrease. The negative effects come from the inherent ecological characteristics and land agglomeration effect of industrial development. Industrial land use itself is a process of consuming resources and discharging waste, thus inevitably exerting negative impacts on the eco-environment. As a result of industrial development, areas with higher returns are more likely to attract new non-land and land factors. With the process of aggregation of new industrial land, inefficient land use is an important reason for reducing GEILU.

### 2.2. Upgrading of Industrial Structure

UIS also has both positive and negative effects on GEILU. The positive impacts are generated through value-added effect, substitution effect, and technology advancement [19,24]. The negative effects come from the inherent ecological characteristics and land agglomeration effect of industrial development. For the impacts of technology advancement, inherent ecological characteristic, and land agglomeration, effects are similar in the introduction of RIS, and we explain the value-added effect and substitution effect here. According to the theory of industrial economics, UIS comes from the transformation of consumer demand and manifests in the transformation of light industry into heavy industry, technology advancement, and the creation and growth of emerging industries [35]. First, the industrial chain is extended with the development of emerging industries, which can stimulate additional economic benefits of industrial land use and improve the economical utilization of land resources. Second, when emerging industry and heavy industry substitute light industry as the growth pole, the industrial output value often shows an upward trend, which will directly improve economical utilization of land resources [36,37].

### 2.3. Ecologization of Industrial Structure

EIS impacts GEILU by reducing eco-environmental disturbance directly and changing the economical utilization of land resources indirectly. In term of the direct effects, ecologization itself is a process of developing low-pollution and low-consumption industries, which are beneficial to reduce the interference of industrial production to the eco-environment. In term of the indirect effects, EIS helps to accelerate the technology advancement in industrial production, in which the resource utilization efficiency and production efficiency are improved [38]. This has a positive effect on the economical utilization of land resources. However, there is often a trade-off between eco-environment protection and economic development. The restriction on the protection of eco-environment will also hinder the speed of economic development to a certain extent, which will have a negative effect on the economical utilization of land resources.

## 3. Methods and Data

### 3.1. Method for Measuring the Green Efficiency of Industrial Land Use

Data envelopment analysis (DEA) is the most popular approach in various types of efficiency evaluation. Because there is no need to preset functional relationships, it can reduce the influence of subjective factors [39,40]. DEA was proposed by Charne, Cooper, and Rhodes in 1978 as a means of evaluating the relative efficiency of decision-making units [41]. In 2001, Tone proposed a slacks-based measure (SBM) of efficiency in DEA [42]. He solved the problem that the traditional model ignored slacks between input and output. With the help of DEA-SBM, the unexpected output was introduced into the efficiency evaluation, which gradually became the mainstream model used to measure green efficiency. Considering that multiple decision-making units were often completely effective in DEA-SBM, in 2002, Tone proposed a super-SBM (slacks-based measure) of efficiency in DEA based on modified slack variables, which effectively solved the issue of the SBM model not being able to sort completely effective units [43]. Based on the above analysis, this research used the super-SBM model to measure GEILU:

(1) $$\rho =\min\frac{\frac{1}{m}\sum_{i=1}^{m}\bar{x_{i}}/x_{i0}}{\frac{1}{s_{1}+s_{2}}\left(\sum_{r=1}^{s_{1}}{\bar{y}}_{r}^{g}/y_{r0}^{g}+\sum_{j=1}^{s_{2}}{\bar{y}}_{j}^{b}/y_{j0}^{b}\right)}$$

(2) $$s.t.x_{0}=X\lambda +S^{-},y_{0}^{g}=Y^{g}\lambda -S^{g},y_{0}^{b}=Y^{b}\lambda +S^{b}$$

(3) $$\bar{x}\geq \sum_{j=1,\neq 0}^{n}\lambda_{j}x_{j},{\bar{y}}^{g}\leq \sum_{j=1,\neq 0}^{n}\lambda_{j}y_{j}^{g},{\bar{y}}^{b}\leq \sum_{j=1,\neq 0}^{n}\lambda_{j}y_{j}^{b}$$

(4) $$\bar{x}\geq x_{0},{\bar{y}}^{g}\leq y_{0}^{g},{\bar{y}}^{b}\geq y_{0}^{b}$$

(5) $$\sum_{j=1,\neq 0}^{n}\lambda_{j}=1,S^{-}\geq 0,S^{g}\geq 0,S^{b}\geq 0,{\bar{y}}^{g}\geq 0,\lambda \geq 0$$

where *ρ* is the value of GEILU; *x*, *y^g^*, and *y^b^* are input, expected output, and unexpected output, respectively; *n*, *m*, *s*_1_, and *s*_2_ are the number of decision-making units, input indices, expected output indices, and unexpected output indices, respectively; *S*^−^, *S^g^*, and *S^b^* are relaxation of the input, expected output, and unexpected output, respectively; and *λ* is the weight vector. *ρ* ≥ 1 indicates a relatively effective decision-making unit, and *ρ* < 1 indicates relative inefficiency.

Referring to the existing research, we used the following indices while calculating the GEILU: (1) the input includes industrial land area, average annual employees per unit area, and industrial fixed asset investment per unit area; (2) the expected output (economic output) is the gross value of industrial output per unit area; (3) the unexpected output (negative externalities) includes industrial SO_2_ discharge per unit area, industrial soot discharge per unit area, and industrial solid waste discharge per unit area. These three indices were considered as the main negative externalities of enterprise activities in China and were recorded in the ***China Statistical Yearbook***. Per unit area refers to the industrial land area.

### 3.2. Variables for Evaluating the Industrial Structure

Three variables—RIS, UIS, and EIS—were introduced to evaluate OIS according to the analytical framework in Section 2.

#### 3.2.1. Rationalization of Industrial Structure

Based on the findings from Lv and Chen (2016) [33], the deviation degree of industrial structure is a common index that can be used to explain RIS. The higher the deviation degree, the more unreasonable the allocation of production factors, the lower the RIS, and the greater the likelihood of an uneven tendency of economic development and vice versa. This research considered labor and capital to be important factors in production and evaluated RIS as follows:

(6) $$D={\left\{\prod_{j=1}^{2}\left[\sum_{i=1}^{n}(\frac{G_{i}}{G})\sqrt{{\left(\frac{G_{i}/G}{P_{ij}/P_{j}}-1\right)}^{2}}\right]\right\}}^{\frac{1}{2}},R=\frac{1}{D}$$

where *R* is the coefficient of RIS, *D* is the deviation degree, *G* is the total output value of industrial land, *G_i_* is the output value of industrial category *i*, *P_j_* is the total investment of production factors *j*, and *P_i_*_,*j*_ is the investment of production factors *j* on industrial category *i*.

#### 3.2.2. Upgrading of Industrial Structure

Based on the findings from Lv and Chen (2016) [33], this research used the following formula to calculate the degree of UIS:

(7) $$U={\left[\prod_{j=1}^{2}(\sum_{i=1}^{n}\frac{G_{i}}{G}\times \frac{G_{i}}{P_{ij}})\right]}^{\left(\frac{1}{2}\right)}$$

where *U* is the coefficient of UIS. The explanation of other variables is the same as the introduction of RIS.

#### 3.2.3. Ecologization of Industrial Structure

According to the definition of eco-environmental protection industry, some studies use the proportion of output value of eco-environmental protection industry to evaluate EIS. However, this method is not conducive to fully reflect the eco-environment characteristics of various industries. This study is based on a certain subjective ranking of industrial categories and used the hierarchical method to evaluate EIS [38]:

(8) $$E=\sum_{j=1}^{n}\sum_{i=1}^{j}\frac{G_{i}}{G}$$

where *E* is the coefficient of EIS, *G*_1_ is the output value of the highest-level industry that has the least damage to eco-environment, and *G_n_* is the output value of the lowest-level industry that has the biggest damage to eco-environment. The greater the value of *E*, the more conducive the industrial structure is to eco-environmental protection.

### 3.3. Method for Measuring the Spatial Effects

LeSage and Pace (2009) constructed the spatial Durbin model (SDM), which includes spatial lag terms of independent variables and dependent variables and has advantages in the investigation of spatial effects [44]. Considering the spatial interaction in industrial structure and land use [45], this research used SDM to analyze the spatial effects of OIS on GEILU. The model was as follows:

(9) $$\begin{array}{l} \ln\rho_{it}=\alpha +\delta W_{ij}\ln\rho_{it}+\beta_{1}\ln U_{it}+\beta_{2}\ln R_{it}+\beta_{3}\ln E_{it}+\gamma_{1}\ln PD_{it}+\\ \gamma_{2}\ln GDPPC_{it}+\gamma_{3}\ln WPC_{it}+\gamma_{4}\ln STID_{it}+\gamma_{5}\ln OOW_{it}+\theta_{1}W_{ij}\ln U_{it}+\\ \theta_{2}W_{ij}\ln R_{it}+\theta_{3}W_{ij}\ln E_{it}+\psi_{1}W_{ij}\ln PD_{it}+\psi_{2}W_{ij}\ln GDPPC_{it}+\psi_{3}W_{ij}\ln WPC_{it}\\ +\psi_{4}W_{ij}\ln STID_{it}+\psi_{5}W_{ij}\ln OOW_{it}+\mu_{i}+\nu_{t}+\ln\varepsilon_{it} \end{array}$$

where *PD* (population density: urban population/urban construction land area, unit: 10^4^ people/km^2^), *GDPPC* (GDP per capita: GDP of 2nd and 3rd industry/urban population, unit: 10^4^ CNY/people), *WPC* (wage per capita of urban workers, unit: CNY), *STID* (science and technology investment density: expenditure for science and technology/general public budget expenditure, unit: %), and *OOW* (opening to the outside world: total value of exports and imports/GDP, unit: %) are control variables, and *ρ* is dependent variable; *R*, *U*, and *E* are independent variables; *I* and *j* are research units, *t* is year, *W* is spatial adjacent weight matrix of 31 research units, *δ* represents spatial lag coefficient, *β* represents regression coefficient of independent variable, *γ* represents regression coefficient of control variable, *θ* and *Ψ* are regression coefficients of corresponding spatial lag terms, *α* is constant, *μ* is spatial fixation effect, *ν* is time fixation effect, and *ε* is random error.

Unlike the traditional non-spatial model, it is worth noting that the variable’s marginal effect cannot be directly obtained from the coefficient of estimation results in above SDM. To address this problem, our research further carried out a spatial effect decomposition analysis using the partial differential decomposition method [46]. In the decomposition results of spatial effects, direct effect quantifies the effect of local independent variables on local dependent variables, indirect effect quantifies the effect of local independent variables on adjacent dependent variables, and total effect is the effect of local independent variables on overall dependent variables.

### 3.4. Data Source

#### 3.4.1. Study Area

**Figure 3:**
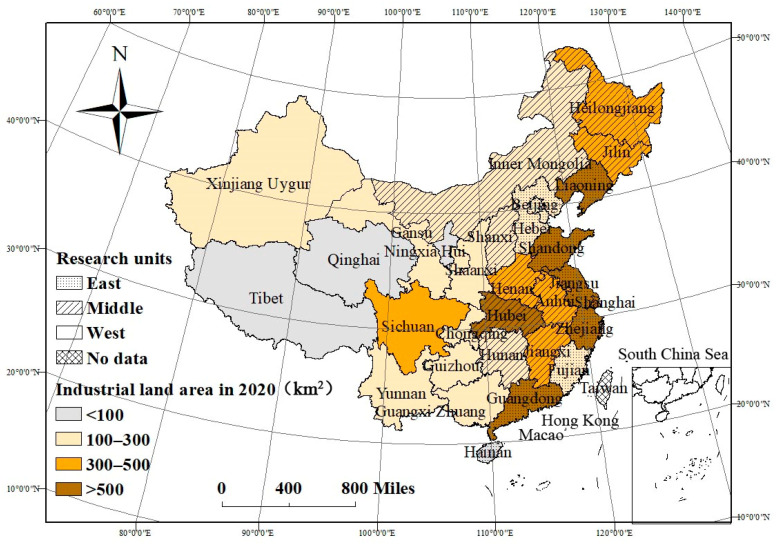
The distribution and industrial land area of research units. Note: The statistics were collected from the ***China Urban Construction Statistical Yearbook***.

Provincial governments in China have the power to create industrial policies and can, to some extent, redistribute public administrative power and resources, which leads to the categorization and spatial distribution of local industrial land. This research took 31 provincial-level administrative regions as research units and analyzed data that emerged from 2006 to 2020 under the consideration of availability of data. There were obviously spatial ladder characteristics in the development of research units. The eastern region had been developed for a longer period of time and was populous and economically successful. The western region was ecologically fragile, impoverished, and relatively sparsely populated, while the middle region was in a transition period that situated it between east and west. Hong Kong, Macao, and Taiwan were not considered in this study due to their different political systems. The distribution of research units is depicted in Figure 3. There were obvious differences in the industrial land area to show that it was decreased from the eastern region to the western region in 2020.

#### 3.4.2. Data and Processing

The definition that we use for industrial land originates from the ***Classification of Urban Land Use and Planning Standards of Development Land***, published by China’s Ministry of Housing and Urban-Rural in 2011, which divided urban land into eight different types. Information about industrial land area and urban construction land area was collected from the ***China Urban Construction Statistical Yearbook*** (2006–2020). The data on regional GDP, gross value of industrial output, industrial fixed asset investment, average number of annual employees in industry, urban population, per capita wage of urban workers, expenditure for science and technology, general public budget expenditure, total value of exports and imports, industrial SO_2_ discharge, industrial soot discharge, and industrial solid waste discharge were gathered from the ***China Statistical Yearbook*** (2007–2021).

In the process of evaluating OIS, category and ranking of industries need to be defined. According to the ***China Industry Statistical Yearbook***, there are three primary categories and 41 subcategories of industries that use the industrial land in China (Table 1). Considering that this kind of classification is not conducive to analyzing the economy and ecology of industries, we did not use it directly. Just as the Chinese government does while accelerating the development of emerging, innovative, and green industries and eliminating low-efficiency and high-pollution industries, the subcategories were reclassified into five categories, which were ranked from low to high as resource-intensive industries, labor-intensive industries, energy-intensive industries, capital-intensive industries, and technology-intensive industries (Table 1) [47]. We expected the ecologization of industrial categories to increase in turn.

The calculations of *R*, *U*, and *E* were based on the refreshed category and ranking. The output value, fixed asset investment, and average number of annual employees of industries in refreshed categories were obtained by summing up what is in corresponding subcategories. The related data of industries in subcategories were gathered from the ***China Industry Statistical Yearbook*** (2007–2021). For some data missing in 2017 and 2018, we estimated with regressions and predictions.

In order to eliminate the impact of price changes, the data linked to industrial output value, fixed asset investment, and GDP were converted into constant prices in 2006 according to the corresponding price indices.

## 4. Results and Discussions

**Figure 4:**
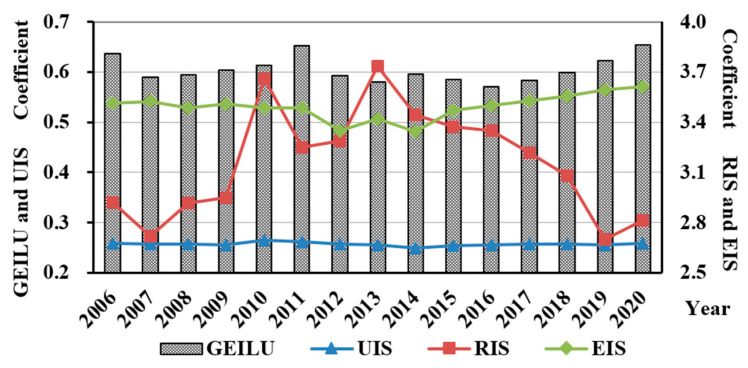
The annual average values of GEILU, RIS, UIS, and EIS.

**Figure 5:**
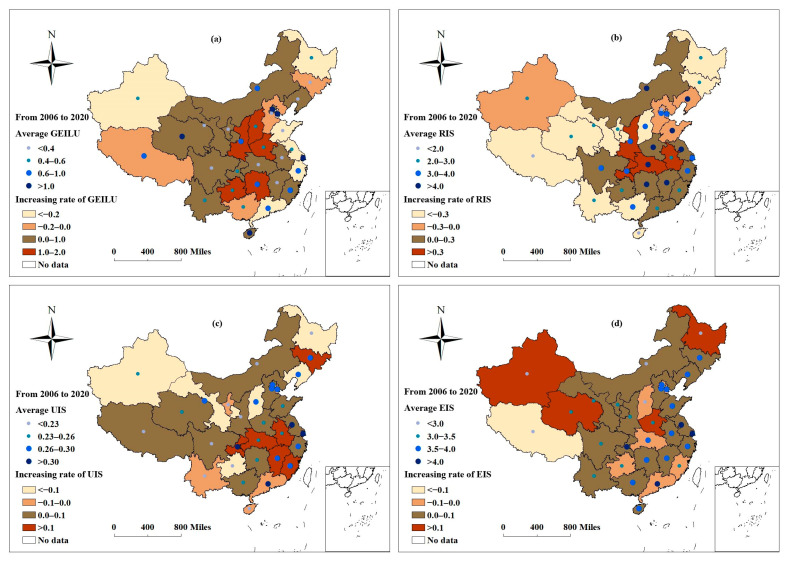
The average GEILU, RIS, UIS, and EIS of research units and the increasing rates from 2006 to 2020. (**a**–**d**) shows the average GEILU, RIS, UIS, and EIS of each research unit and the increasing rates from 2006 to 2020, respectively.

The coefficient of GEILU was measured using the super-SBM model. The coefficients of UIS, RIS, and EIS were calculated using Formulas (6) through (8). Figure 4 shows the annual average value of GEILU, RIS, UIS, and EIS. Figure 5a–d shows the average GEILU, RIS, UIS, and EIS of each research unit and the increasing rates from 2006 to 2020, respectively.

### 4.1. Analysis of the Green Efficiency of Industrial Land Use

The annual average value of GEILU shows a wave change from 2006 to 2020 and kept increasing after 2016 (Figure 4). By tracing the original evaluation indices, the sustained increase from 2016 to 2020 was the result of the reduction in pollutant emissions.

Figure 5a shows the average GEILU of each research unit. The higher values were mostly distributed in the east, while the lower values were mostly distributed in middle and northern China. The average GEILU of several western provinces was at a higher level. The western provinces were typically ecologically fragile areas, and regional industrial development was restricted by ecological protection policies. Therefore, the negative external ecological effect of industrial land use was relatively weak.

The increasing rates of GEILU of the 31 research units are depicted in Figure 5a. The GEILU of 11 units decreased to different degrees and was concentrated in the eastern and western region. It was inferred that these provinces did not create a balance between ecological benefits and rapid economic development; however, the middle provinces had a visible improvement in low-pollution and highly efficient industrial land-use patterns.

### 4.2. Analysis of Industrial Structure

For the statistics of annual average values from 2006 to 2020, Figure 4 shows that there was almost no change in the UIS, but the RIS and EIS changed in a positive U shape and an inverted U shape, respectively. The apexes of the U shape corresponded to around 2013, indicating that the industrial structure was developing towards ecologization from 2013, but the production factors such as labor and capital were distributed in an unreasonable way.

Figure 5b–d shows the average RIS, UIS, and EIS of each research unit and the increasing rates from 2006 to 2020. The provinces with higher average RIS were distributed in the middle and east, while the lower values were mostly distributed in the west. The RIS of 16 provinces, which were concentrated in the northeastern and western provinces, were declining, and four provinces, most of which located in central China, maintained a high growth rate of more than 30% (Figure 5b). The average UIS descended in the east to the west, too. The changes of UIS showed that the UIS in central China also increased more rapidly than it did in western China (Figure 5c). Moreover, the average EIS presented a more obvious step feature that showed that high-value units were concentrated in the prosperous east and that low-value units were concentrated in the relatively poor west (Figure 5d). Some resource-based units, such as Tibet, Heilongjiang, Inner Mongolia, Xinjiang, and Shanxi, had the lowest value of EIS. However, changes in EIS showed some randomness in space. It was found that some resource-based units, such as Fujian, Tibet, and Guizhou, also maintained a decline rate of more than 10%, but other resource-based units, such as Xinjiang and Qinghai, experienced a greater adjustment of EIS and maintained an increasing rate of more than 20%.

### 4.3. Analysis of the Spatial Effects

#### 4.3.1. Estimation Results

SDM was used to capture the spatial effects of RIS, UIS, and EIS on GEILU. First, the LR test, Ward test, and Hausman test were conducted to determine the specific form of SDM [46]. The result shows that they all passed the significance-level test of 1%, indicating that spatial lag and spatial error exist simultaneously; thus, the SDM with fixed effect was selected for model regression.

There are three types of fixed-effect models for space fixed effect, time fixed effect, and bidirectional fixed effect. Judging by a further LR test [46], the spatial effects were simulated using a SDM with bidirectional fixed effect, the results of which are shown in Table 2 as model (1).

The regression coefficient of Ln*R* was positive, which suggested that RIS had a promotion effect on GEILU. In the process of RIS, industrial land had a more efficient allocation, which was suitable for regional comparative advantages. Different production factors were substituted to earn the most profit and contributed to maximizing the output value of industrial land use. The spatially weighted upgrade term W×Ln*R* had a significant positive regression coefficient, indicating that the improvement of RIS in surrounding regions drove the rational allocation of local industrial land and production factors and ultimately improved the local GEILU.

The regression coefficient of Ln*U* was negative, which suggested that UIS had an inhibiting effect on GEILU. This result may stem from the fact that China was in the mid-term of industrialization in which the industrial structure transformed from light textile industry to heavy chemical industry. The industrial categories including automobile, communication, and infrastructure construction were growth poles in the industrial economy that drove the development of iron, steel, machinery, and chemical industry. As high-consumption, high-pollution, and high-emission industries exited the market, UIS improved the economical utilization of industrial land but also increased the disturbance to eco-environment and eventually created a negative effect on GEILU. The results showed that the regression coefficient of the spatially weighted upgrade term W×Ln*U* was 0.553, but it was not significant, which proved that UIS had no obvious spatial effect on GEILU.

The regression coefficient of Ln*E* was negative, suggesting that EIS had an inhibiting effect on GEILU. The inefficient expansion of industrial land area was a serious problem in the research period, which damaged the economic utilization of industrial land to a certain extent. In addition, influenced by the government’s obsessive concentration on GDP growth, industrial enterprises increased their output through high-consumption rather than technological advancement. Outdated technology led to the uncontrollable emission of industrial pollutants. The spatially weighted upgrade term W×Ln*E* had a significant negative regression coefficient as well. The improvement of EIS in the surrounding areas led to the agglomeration of production factors, which resulted in the loss of local production factors and thus inhibited the effective utilization of local industrial land.

#### 4.3.2. Robust Test of the Estimation Results

In order to test whether the estimation results changed with the parameter setting, we conducted robust tests using the following approaches [32]:

(1) Re-evaluation of GEILU. We calculated the GEILU using the super-SBM model under the restriction that the variable returned to scale and then captured the effects of RIS, UIS, and EIS on GEILU, leaving the other conditions unchanged. The estimation results are shown in Table 2 as model (2).
(2) Transformation of spatial weight matrix. We replaced the spatial adjacent weight matrix with an inverse-distance space weight matrix and then captured the effects of RIS, UIS, and EIS on GEILU, leaving the other conditions unchanged. The estimation results are shown in Table 2 as model (3).

It was determined that the effect direction and significance of the dependent variables in the three models had not changed significantly. The estimation results in this research were therefore robust.

#### 4.3.3. Analysis of the Integration Results of the Three Variables

The direct, indirect, and total effects of Ln*R*, Ln*U*, and Ln*E* were quantified by the spatial effect decomposition analysis and are shown in Table 3.

For the direct effects, the regression coefficients of Ln*R*, Ln*U*, and Ln*E* all passed the significance test. Taking RIS as an example, the regression coefficient of direct effect was 0.005, which suggested that the value of local GEILU increased by 0.005% for every 1% increase in the value of local RIS, leaving the control of other factors unchanged. Ln*U* and Ln*E* showed different degrees of inhibiting effect with coefficients of −0.722 and −0.360, respectively. For the indirect effects, the regression coefficients of Ln*R* and Ln*E* passed the significance test. The value of adjacent GEILU increased by 0.001% for every 1% increase in the value of local RIS and declined by 0.224% for every 1% increase in the value of local EIS, leaving the control of other factors unchanged. The regression coefficient of the total effect was the sum of that of direct effect and indirect effect.

The integrated effects of OIS on GEILU were analyzed based the sum of the regression coefficients of Ln*R*, Ln*U*, and Ln*E*. The summarized coefficients of direct effect, indirect effect, and total effect were −1.077, 0.328, and −0.749, respectively. It was determined that OIS had a significantly negative effect on GEILU during the study period. For the local effect, OIS helped improve the output value of industrial land use; however, it also led to an inefficient expansion of industrial land and pollution from heavy chemical industries, which resulted in a negative effect on GEILU. For the spatial effect, the cooperation with adjacent provinces played a positive role in improving GEILU. Although the competition for production factors from adjacent provinces reduced the local GEILU, it was not enough to offset the effects of cooperation.

According to the above analysis on the integrated effects of OIS on GEILU, this research determined that provincial governments, especially those in eastern China with rapid UIS, ascending EIS, and descending GEILU, should support intensive utilization of industrial land and rapid development of high-knowledge and high-tech industries. More flexible land supply policies should be formulated that are conducive to the development cycle of emerging and traditional industries, such as combining short-term and long-terms land leasing. More efforts should also be made to redevelop inefficient industrial land. Furthermore, spatial effects should be considered during the creation of industrial land policies. Coordinating the use of industrial land from a macro-scale contributed to eliminating the market segmentation between provinces, thus improving the green and highly efficient use of industrial land.

### 4.4. Analysis of Threshold Effect

**Figure 6:**
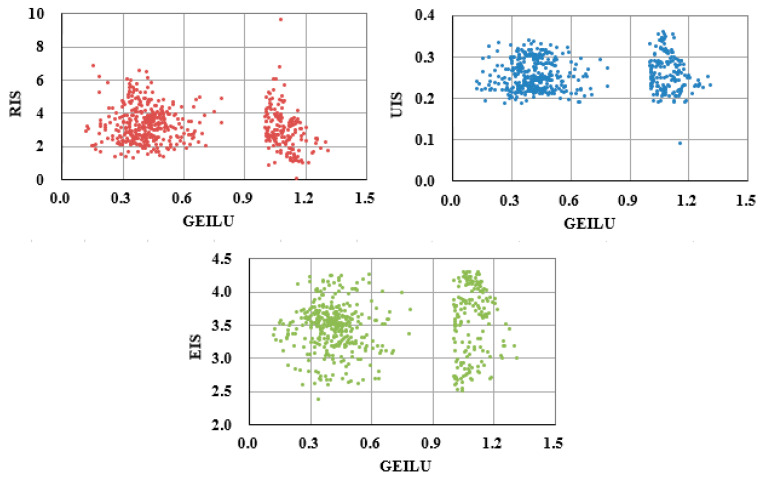
The scatter diagrams of GEILU, RIS, UIS, and EIS.

Figure 6 depicts scatter diagrams of RIS, UIS, EIS, and GEILU. There were obvious signs of stratification in the range where the value of GEILU was around 0.9, which indicated that there were probably threshold effects between OIS and GEILU [48].

Considering that GDP was a representative index of economic and social development, the control variable *GDPPC* was selected as a threshold variable. The threshold effect was tested using the following panel threshold model:

(10) $$\rho_{it}=\alpha_{i}+\delta X_{it}+\beta_{1}M_{it}I(C_{it}\leq \varphi_{1})+\beta_{2}M_{it}I(\varphi_{1}\leq C_{it}\leq \varphi_{2})+\cdots +\beta_{n+1}M_{it}I(C_{it}>\varphi_{n})+\varepsilon_{it}$$

where *ρ* is dependent variable, *M* is a core independent variable affected by threshold variable, *X* represents a group of variables other than *M* that have a significant effect on the independent variable, *C* is threshold variable, *δ* and *β* are regression coefficients of corresponding variables, *i* is research unit, *t* is year, *I* is exponential function, *φ* is threshold value, *α* is constant, and *ε* is random error.

This research determined the value of *n* through the threshold effect test and then estimated the values of *φ*. Ln*R*, Ln*U*, and Ln*E* were taken as core dependent variables, respectively. The results showed that ln*GDPPC* had double-threshold effects on the three core dependent variables. The double-threshold values, which were 0.581 and 0.648, fell within the interval of (0.496, 0.548) and (0.548, 0.658) under the 95% confidence level, indicating that the threshold had passed the effectiveness test.

The estimation results of the threshold effects are presented in Table 4. For core dependent variable Ln*R*, when ln*GDPPC* was less than 0.581, its regression coefficient was −0.201; when ln*GDPPC* was greater than 0. 581 and less than 0.648, the regression coefficient as −0.102; and when ln*GDPPC* was greater than 0.648, the regression coefficient was 0.206. The three coefficients were all significant. This result showed that under the constraint of *GDPPC*, the relationship between RIS and GEILU presented the characteristics of positive “U”; that is, with the increase of *GDPPC*, the negative effect of RIS on GEILU was weakened and gradually changed into a positive effect.

The regression coefficients of the core dependent variable ln*U* were −2.860, −1.026, and −0.310 with the increase of ln*GDPPC*. The first and second coefficients were significant at the level of 1%, but the third coefficient was not significant. The regression coefficients of the core dependent variable ln*E* were −0.345, −0.164, and 0.115 with the increase of ln*GDPPC*. Only the second coefficient was not significant. These results indicated that UIS and EIS had significantly negative effects on GEILU at the early stage, but with the value of *GDPPC* exceeded certain thresholds, the effects were decreased or even turned positive.

Based on the above analysis of threshold effects, it was speculated that in the early period of this study, outdated technology and economic development caused low-value-added, high-consumption, high-pollution, and high-emission industries to exit the market. As a result, OIS had negative effects on GEILU; however, with industrial and economic development, high-knowledge and high-tech industries created a market scale, and the negative effects gradually shifted into positive or insignificant. Therefore, industrial land-use policies should change according to the different stages of industrial development. Provinces with traditional industrial structure face more serious waste of resources and ecological pollution. Strengthening intensive land use and eco-environmental control helped improve GEILU in these cases; however, for provinces with rapid development of emerging industries, OIS contributed to the improvement of GEILU. More attention should be given to the differentiated supply policies of industrial land that is used by high-knowledge and high-tech industries.

## 5. Conclusions

Improving GEILU is essential to promoting low-pollution and highly efficient development. Considering that industrial structure is a key factor that affects the green efficiency, this paper focused on revealing whether or not OIS improves GEILU in China, using the panel data of 31 provincial units collected between 2006 and 2020. Based on an analytical framework for exploring the effect mechanisms from the perspective of rationalization, upgrading, and ecologization of industrial structure, the GEILU was measured using the super-SBM model, while the OIS was evaluated through the three variables of RIS, UIS, and EIS, and the spatial effects of OIS on GEILU were captured using SDM. The results were as follows:

(1) On the national scale, the GEILU showed fluctuating change from 2006 to 2020 and kept increasing after 2016. The eastern provinces presented relatively higher GEILU, but the middle provinces showed significant improvement. There was almost no change in the UIS; however, the RIS and EIS changed in a positive U shape and an inverted U shape, respectively. The apexes of the U shape were both correlated to around 2013.
(2) The regression results of SDM showed that, from a global perspective, the RIS contributed to the improvement of GEILU and that there was a positive spatial effect. The UIS had an inhibiting effect on GEILU but had no obvious spatial effect. The EIS had an inhibiting effect on GEILU, and there was a negative spatial effect. When integrating the three variables, OIS did not contribute to the improvement of GEILU but presented significantly negative effects. The negative effects stemmed from an inefficient expansion of industrial land and pollution from heavy chemical industries. However, the cooperation with adjacent provinces improved GEILU to a certain extent.
(3) From a phased perspective, the effects of OIS on GEILU had double-threshold characteristics. When the increase of GDP per capita exceeded the thresholds, the effects of RIS and EIS on GEILU changed from negative to positive, and the negative effect of UIS gradually eased and became insignificant. It was speculated that in the early period of this study, the outdated technology in traditional industries brought about the negative effects of OIS on GEILU; however, with industrial and economic development, high-knowledge and high-tech industries created a market scale, and the negative effects gradually shifted into positive or insignificant.
(4) In order to improve the GEILU, provinces with traditional industrial structures should bolster intensive land use and eco-environmental control; however, provinces with rapid development of emerging industries should support the development of high-knowledge and high-tech industries through creating and implementing more flexible land supply policies. Moreover, eliminating the market segmentation between provinces can also help improve GEILU.

## Figures and Tables

**Table 1:** Industry categories in statistical yearbook and refreshed categories used by this study.

Statistical Categories	Statistical Subcategories	Refreshed Categories
Mining	Coal mining and dressing; oil and gas mining; ferrous metals mining and dressing; non-ferrous metals mining and dressing; non-metallic ore mining and dressing; professional and auxiliary activities; other.	Resource-intensive industries
Manufacturing	Agricultural food processing; food manufacturing; beverage manufacturing; tobacco manufacturing; textiles; leather, fur, feather, and their products; wood processing and related products; furniture manufacturing; clothing manufacturing; papermaking and paper products; printing and recording media reproduction; manufacturing of cultural, educational, artistic, sports and entertainment products; rubber and plastic products.	Labor-intensive industries
	Petroleum processing, coking and nuclear fuel processing; non-metallic mineral products; ferrous metal smelting and rolling processing; nonferrous metal smelting and rolling processing; metal products; general equipment manufacturing; special equipment manufacturing; instrument manufacturing.	Capital-intensive industries
	Chemical raw materials and products manufacturing; pharmaceutical manufacturing; chemical fiber manufacturing; automobile manufacturing; transportation equipment manufacturing; electrical machinery and equipment manufacturing; electronic equipment manufacturing; comprehensive utilization of waste resources; repair of metal products, machinery, and equipment; other.	Technology-intensive industries
Production and supply of power, heat, gas, and water	Production and supply of power and heat; production and supply of gas; production and supply of water.	Energy-intensive industries

Note: Statistical categories and subcategories are derived from the ***China Industry Statistical Yearbook***.

**Table 2:** The estimation results of spatial Durbin model.

Variables	Model (1)		Model (2)		Model (3)	
	Regression Coefficients	*t-Stat*	Regression Coefficients	*t-Stat*	Regression Coefficients	*t-Stat*
Ln*ρ*	0.332 ***	(6.39)	0.160 ***	(2.68)	0.197 ***	(3.29)
Ln*R*	0.005 **	(0.17)	0.015 *	(0.24)	0.068 **	(2.04)
Ln*U*	−0.822 *	(−1.35)	−0.675 *	(−0.82)	−0.067	(−0.15)
Ln*E*	−0.348 *	(1.93)	−0.016	(0.05)	−0.460 **	(2.43)
Ln*PD*	0.296 ***	(5.04)	0.128	(1.12)	0.209 ***	(3.86)
Ln*GDPPC*	0.421 ***	(6.63)	0.532 ***	(4.26)	0.154 **	(2.58)
Ln*WPC*	0.551 ***	(8.02)	0.478	(3.55)	0.048	(0.76)
Ln*RDID*	−0.128 *	(−1.37)	−0.473 **	(−2.57)	−0.035	(−0.35)
Ln*OOW*	0.342	(0.65)	−0.790	(−0.77)	0.993 **	(2.50)
W×Ln*R*	0.001 *	(0.06)	0.438 ***	(4.28)	0.055	(0.99)
W×Ln*U*	0.553	(0.72)	0.591	(0.39)	−0.172	(−0.23)
W×Ln*E*	−0.254 **	(−0.73)	−1.588	(−2.33)	−0.970 **	(−2.52)
W×Ln*PD*	−0.384 ***	(−4.99)	−0.382 **	(−2.52)	−0.254 ***	(−3.51)
W×Ln*GDPPC*	−0.379 ***	(−4.37)	−0.589 ***	(−3.45)	−0.143	(−1.45)
W×Ln*WPC*	−0.650 ***	(−8.26)	−0.695 ***	(−4.50)	−0.054	(−0.63)
W×Ln*RDID*	−0.035	(−0.24)	0.214	(0.73)	−0.026	(−0.16)
W×Ln*OOW*	−0.162	(−0.31)	0.961	(0.93)	−0.849 **	(−2.08)
R^2^	0.145		0.131		0.086	
log-likelihood	425.396		75.701		268.974	

Note: *, **, and *** indicate the level of significance at 10%, 5%, and 1%, respectively.

**Table 3:** The decomposition results of spatial effects.

Variables	Direct Effect		Indirect Effect		Total Effect	
	Regression Coefficients	*t-Stat*	Regression Coefficients	*t-Stat*	Regression Coefficients	*t-Stat*
Ln*R*	0.005 *	(0.16)	0.001 *	(0.01)	0.006 *	(0.07)
Ln*U*	−0.722 **	(−2.18)	0.551	(0.96)	−0.171	(0.21)
Ln*E*	−0.360 *	(1.63)	−0.224 **	(−0.73)	−0.584 **	(−2.01)
Ln*PD*	0.309 ***	(4.60)	−0.357 **	(−4.36)	−0.048 **	(−0.37)
Ln*GDPPC*	0.425 ***	(5.49)	−0.282 ***	(−4.37)	0.143 ***	(3.86)
Ln*WPC*	0.537 ***	(7.68)	−0.598	(−8.32)	−0.061	(−0.98)
Ln*RDID*	−0.154 *	(−1.05)	−0.048	(−0.34)	−0.202	(−1.36)
Ln*OOW*	0.169	(0.31)	−0.086	(−0.21)	0.083	(0.16)

Note: *, **, and *** indicate the level of significance at 10%, 5%, and 1%, respectively.

**Table 4:** Estimation results of threshold effect.

Threshold Values	Ln*R*		Ln*U*		Ln*E*	
	Regression Coefficients	*t-Stat*	Regression Coefficients	*t-Stat*	Regression Coefficients	*t-Stat*
ln*GDPPC* ≤ 0.581	−0.201 ***	(−4.26)	−2.860 ***	(−5.15)	−0.345 *	(−1.89)
0.648 ≤ ln*GDPPC* ≤ 0.581	−0.102 **	(−1.96)	−1.026 ***	(−2.48)	−0.164	(−0.78)
ln*GDPPC* ≥ 0.648	0.206 ***	(3.08)	−0.310	(−0.62)	0.115 *	(0.31)

Note: *, **, and *** indicate the level of significance at 10%, 5%, and 1%, respectively.

## Data Availability

The datasets generated during and/or analyzed during the current study are available from the corresponding author on reasonable request.
